# An FPT haplotyping algorithm on pedigrees with a small number of sites

**DOI:** 10.1186/1748-7188-6-8

**Published:** 2011-04-19

**Authors:** Duong D Doan, Patricia A Evans

**Affiliations:** 1 Faculty of Computer Science, University of New Brunswick, Fredericton, New Brunswick, Canada

## Abstract

**Background:**

Genetic disease studies investigate relationships between changes in chromosomes and genetic diseases. Single haplotypes provide useful information for these studies but extracting single haplotypes directly by biochemical methods is expensive. A computational method to infer haplotypes from genotype data is therefore important. We investigate the problem of computing the minimum number of recombination events for general pedigrees with a small number of sites for all members.

**Results:**

We show that this NP-hard problem can be parametrically reduced to the Bipartization by Edge Removal problem with additional parity constraints. We solve this problem with an exact algorithm that runs in  time, where *n *is the number of members, *m *is the number of sites, and *k *is the number of recombination events.

**Conclusions:**

This algorithm infers haplotypes for a small number of sites, which can be useful for genetic disease studies to track down how changes in haplotypes such as recombinations relate to genetic disease.

## Background

Human genomes contain two copies of each chromosome. Research shows that single chromosomes, called haplotypes, are useful to study complex genetic diseases. While genomic data, called genotypes, are abundant and easy to collect, haplotypes are rare and much more difficult to obtain by a biochemical method. Therefore, computationally inferring haplotypes from genotype data, called haplotyping, is necessary. Genotypes can be obtained from a population group where relationships between members are unknown or from a family pedigree with known relationships between members. We only consider pedigree data.

In the absence of recombination events, haplotypes of members in a pedigree follow the Mendelian law of inheritance, where the two haplotypes of a child are transferred from its parents, one haplotype from its father and the other from its mother. Various haplotyping algorithms exist for non-recombinant pedigree data [1,2], especially a linear algorithm for tree pedigrees [1] and a near-linear algorithm for general pedigrees [2]. Haplotype inference is complicated by recombination events and the complex structures of the data. In recombination events, complementary parts of both of a parent's haplotypes can be inherited as a single combined haplotype of a child. Structures of the pedigree can be complex, where there are multiple inheritance paths between some family members.

When recombination events are allowed, the problem of inferring haplotypes for pedigrees with the minimum number of recombination events is NP-hard, even for general pedigrees with only two sites or tree pedigrees with multiple sites [3]. For reconstructing haplotype configurations for pedigree data, Qian and Beckmann [4] proposed a rule-based algorithm with a time complexity *O*(2*^d^n*^2^*m*^3^), for *n *members, *m *sites, and family size ≤ *d*. The main principle of their algorithm is that the best haplotype configuration for pedigree data is the one that minimizes the number of recombination events (the *MRHC problem*). Li and Jiang [5] proposed an integer linear programming (ILP) formulation for the MRHC problem. When the number of recombination events is strictly smaller than a positive number *k*, an *O*(*mn *· log^*k*+1 ^*n*) time probabilistic algorithm is given on tree pedigrees [6]. Doan and Evans [7] presented an *O*(2*^k ^*· *n*^2^) time fixed-parameter algorithm for general pedigrees where each member has two sites, a special case of the problem that is still NP-complete.

We study the haplotype inference for general pedigrees with recombination events when the number of recombination events *k *and the number of sites *m *in an input pedigree are small. We also assume that there are no data missing and no data errors. We prove that our problem can be reduced to the problem of finding the *line index *of a *signed graph *[8] with additional parity constraints. We further show that finding the line index of a signed graph can also be reduced to the Graph Bipartization by Edge Removal (*GBER*) problem with parity constraints. The GBER problem is fixed-parameter tractable, but the existing solution [9] cannot satisfy the additional parity constraints. We present an algorithm that solves the problem while still satisfying the additional constraints, and thus show that the Recombinant Haplotype Configuration problem can be solved by a fixed-parameter algorithm with a running time of , for *n *members, *m *sites, and *k *recombination events. This result extends our prior work for pedigrees with two sites to an arbitrary small number of sites.

## Preliminaries

A member is an individual. A set of members is called a *family *if it includes only two parents and their children; it is a *parent*-*offspring trio *(hereafter a *trio*) if only two parents and one child are considered. A set of families connected through known family relationships is a *pedigree*.

In diploid organisms, a cell contains two copies of each chromosome. The description data of the two copies are called a *genotype *while those of a single copy are called a *haplotype*. A specific location in a chromosome is called a *sit*e and its state is called an *allele*. There are two main types of sites, *microsatellites *and *single nucleotide polymorphisms*. A microsatellite site has several different states while a single nucleotide polymorphism (*SNP*) site has exactly two possible states, denoted by 0 and 1. Only SNPs with two possible states are considered in this paper, as in other works on haplotype inference.

If the states at a specific site in two haplotypes are the same, then this site is a *homozygous *site (0-0 or 1-1); if they differ, it is *heterozygous *(0-1 or 1-0). Two haplotypes combine together to form one genotype. Each member *u *has two haplotypes, denoted by *h*1*_u _*and *h*2*_u_*, which are vectors of 0 and 1's of length *m*, where *m *is the number of sites. The genotype of *u*, *g_u_*, is a vector of 0's, 1's and 2's of length *m*, where *g_u_*[*i*] = 0 means *h*1*_u_*[*i*] = 0 = *h*2*_u_*[*i*], *g_u_*[*i*] = 1 means *h*1*_u_*[*i*] = 1 = *h*2*_u_*[*i*], and where *g_u_*[*i*] = 2 means {*h*1*_u_*[*i*]; *h*2*_u_*[*i*]} = {0, 1}. We say *h*1*_u _*and *h*2*_u _*are consistent with *g_u_*. The complement haplotype of a haplotype *h *at a heterozygous site is denoted by , where  so,  and .

**Figure 1:**
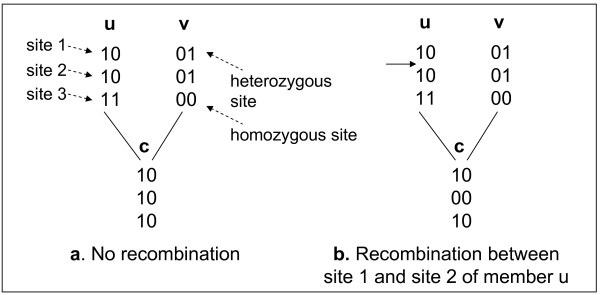
**Non-recombination vs. recombination, showing haplotypes of members**.

When there is no recombination event in a pedigree, a child member receives one entire haplotype from its father and another entire haplotype from its mother. Figure 1a shows member *c *receiving the entire left haplotype of parental member *u *and the entire left haplotype of parental member *v*. However, during the meiosis process, haplotypes of a parent sometimes shuffle due to the crossover of chromosomes and one of the shuffled copies is transferred to the child. This phenomenon is called a recombination and the result is called a recombinant. Figure 1b shows a recombination event between site 1 and site 2 of member *u*. As the result, member *c *receives a combined haplotype from site 1 of the left haplotype, and from sites 2 and 3 of the right haplotype of member *u*.

The problem in this paper is to find the haplotypes *h*1*_u _*and *h*2*_u _*for all members *u *that minimize the number of recombination events, given their genotypes *g_u_*. A set of haplotypes found for all members is called a *haplotype configuration*. When *g_u_*[*i*] = 0 or 1, then *h*1*_u_*[*i*] and *h*2*_u_*[*i*] are known, but if *g_u_*[*i*] = 2, we may not yet know the value of *h*1*_u_*[*i*] and *h*2*_u_*[*i*], in which case we give them the value "?", and say that the site is *unresolved*. Our problem is defined as follows.

**RHC***_opt_*: *Given the genotypes of a general pedigree P containing n members, where each member has m sites (m is small), find a haplotype configuration that minimizes the number of recombination events*.

This optimization problem, called *Recombination Haplotype Configuration *(RHC*_opt_*) which is identical to MRHC, was proven NP-hard [3]. We investigate the corresponding decision version of RHC*_opt_*.

**RHC***_k_*: *Given positive integers k and the genotypes of a general pedigree P containing n members, where each member has m sites (m is small), is there a haplotype configuration with at most k recombination events explaining P ?*

In this paper, we use *u*, *v *and *c *to represent members, from 1 to *n*; and *i *and *j *to represent sites, from 1 to *m*.

## Setting Up Graphs

**Figure 2:**
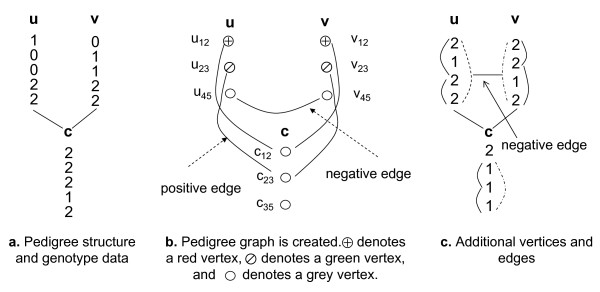
**Pedigree graph created from pedigree structure and genotype data**.

Given a general pedigree with *n *members, where each member has *m *sites, we set up a pedigree graph *G *= (*V*, *E*) and parity-constraint sets *S_pc _*to compute the minimum number of recombination events in the pedigree. A recombination event can only be detected if there is at least one heterozygous site on each side of a recombination breakpoint, e.g. we cannot detect if a recombination event happens between homozygous sites 1 and 3 of member *u *in Figure 2a because the states at the two haplotypes for each homozygous site are the same. The graph captures constraints between pairs of closest heterozygous sites and pairs of closest homozygous sites, which will enable the detection of possible recombination events in pedigrees. A vertex in the pedigree graph represents a pair of homozygous sites or a pair of heterozygous sites, and is colored to represent the relationship between the haplotypes of the sites.

### Pedigree Graph

#### Create grey vertices

Let *i *be a heterozygous site in a member *u *(*i *= 1, ..., *m *- 1). Let *j *>*i *be the closest heterozygous site to *i *in *u*. We create a vertex *u_ij _*from site *i *and site *j *and label this vertex *grey*. A grey vertex is an *unresolved vertex *and will later be resolved green if *h1_u_*[*i*] = *h*1*_u_*[*j*] = 0 or *h*1*_u_*[*i*] = *h*1*_u_*[*j*] = 1. It is resolved red otherwise. The resolution of a grey vertex depends on its adjacent vertices. Figure 2b shows a grey vertex *u*_45 _created from sites 4 and 5 of *u *in Figure 2a.

#### Create red and green vertices

Let *i *be a homozygous site in a member *u *(*i *= 1,..., *m *- 1). Let *j *>*i *be the closest homozygous site to *i *in *u*. We create a vertex *u_ij _*from site *i *and site *j*, and label this vertex *red *if *g_u_*[*i*] ≠ = *g_u_*[*j*] and *green *if *g_u_*[*i*] = *g_u_*[*j*]. A red or green vertex is a *resolved vertex*. Figure 2 shows a red vertex *u*_12 _created from sites 1 and 2, and a green vertex *u*_23 _from sites 2 and 3.

#### Insert positive edges

We insert positive edges between a parent member *u *and its direct child member *v*. For each vertex *u_ij _*in *u*, if there is a vertex *v_ij _*in *v *we insert a *positive edge *between *u_ij _*and *v_ij_*. If there is no vertex *v_ij _*in *v *and *i *and *j *are both homozygous sites or both heterozygous sites in *v*, we create a vertex *v_ij _*in *v *and label this vertex properly, inserting a positive edge between *u_ij _*and *v_ij_*. We call *v_ij _*a *supplementary vertex *as it is created by the need of member *u*.

Similarly, for each vertex *v_ij _*in *v*, if there is no vertex *u_ij _*in *u*, and *i *and *j *are both homozygous sites or both heterozygous sites in *u*, we create a supplementary vertex *u_ij _*in *u *and label this vertex properly, inserting a positive edge between *u_ij _*and *v_ij_*. Figure 2b shows four positive edges linking *u*_12 _and *c*_12 _that is created from heterozygous sites 1 and 2 of member *c*, *u*_23 _and *c*_23_, *v*_12 _and *c*_12_, *v*_23 _and *c*_23_.

A positive edge between vertices *u_ij _*and *v_ij _*means vertex *u_ij _*and *v_ij _*should be resolved with the same color (both red or both green) unless a recombination event occurs in *u*. The reason for this is that if there is no recombination event in *u*, then *v *receives one full haplotype from *u *and another full haplotype from another parent. Therefore, the label of *u_ij _*and the label of *v_ij _*should be the same if there is no recombination event; otherwise, there is a recombination event in *u*. If *u_ij _*is a resolved vertex forming from two homozygous sites *i *and *j *and there is a positive edge between *u_ij _*and a grey vertex *v_ij_*, we color *v_ij _*the same as the color of *u_ij_*, since a recombination event at *u_ij _*is not detectable and does not affect the color of *v_ij_*.

#### Insert negative edges

We insert *negative edges *between two parents *u *and *v *of a common child *c*. If *u_ij _*is a vertex in *u *but there is not a vertex *c_ij _*in *c *(sites *i *and *j *are one homozygous and one heterozygous in *c*), two situations happen. If there is a vertex *v_ij _*in *v*, we insert a negative edge between *u_ij _*and *v_ij_*. Otherwise, if there is no vertex *v_ij _*in *v *and *i *and *j *are both homozygous sites or both heterozygous sites, we create a supplementary vertex *v_ij _*in *v *and label it properly. We insert a negative edge between *u_ij _*and *v_ij_*. Similarly, if *v_ij _*is a vertex in *v *but there is not a vertex *c_ij _*in *c*, there are two situations. If there is no vertex *u_ij _*in *u*, and *i *and *j *are both homozygous or both heterozygous, we create a supplementary vertex *u_ij _*in *u*, and insert a negative edge between *u_ij _*and *v_ij_*. Figure 2b shows a negative edge linking *u*_45 _and *v*_45_.

A negative edge between *u_ij _*and *v_ij _*means vertices *u_ij _*and *v_ij _*should be resolved with different colors unless a recombination event occurs in one parent of *c*. This phenomenon can be explained as follows. If there is no recombination event and *u_ij _*and *v_ij _*have the same label (both red or both green), then sites *i *and *j *of *c *must be both homozygous or both heterozygous based on the Mendelian law of inheritance. Because sites *i *and *j *of *c *are one homozygous and one heterozygous, one recombination occurs if *u_ij _*and *v_ij _*have the same label when resolved, but no recombination event occurs if they are resolved differently.

#### Create additional vertices

Consider a grey vertex *u_ij _*in *u *(*i *<*j*). It is possible that *u_ij _*has no incident edge but there is one recombination event occurring between site *i *and *j*. In this case none of the other two members in the trio has a vertex created for site *i *and *j*. We delete vertex *u_ij _*and create an additional vertex to capture the recombination event. Let *j' *be the closest heterozygous site from *j *in *u *(*j *<*j'*), where *i *and *j' *are both heterozygous sites or both homozygous sites in at least one member among the other two members, say *v*. If there is no vertex *u_ij' _*in *u*, we create an *additional *grey vertex *u*_*ij*' _in *u *and create a supplementary vertex *c_ij' _*from sites *i *and *j' *in *c *if it does not exist. We color *c_ij' _*properly and insert a corresponding edge (positive or negative) between *u_ij' _*and *v_ij' _*depending on the relationship between *u *and *v*. Figure 2c shows an additional vertex *u*_14 _created represented by a dashed edge between sites 1 and 4. A negative edge is inserted between *u*_14 _and *v*_14_.

#### Pedigree graph

Pedigree graph *G *= (*V*, *E*) created as described above is an undirected graph. Each vertex *y *∈ *V *has three possible labels, red, green, and grey. Each edge *e*(*y*, *z*) ∈ *E *is either a positive edge, *e *∈ *E_pos_*, or a negative edge, *e *∈ *E_neg_*, with *E *= *E_pos _*∪ *E_neg_*. Graph *G*, set up this way, is a signed graph [8]. Let *N*(*y*) be the set of adjacent vertices of *y*. Let *w*(*e*) be the weight of edge *e*. If *e *is a positive edge, *w*(*e*) = +1. If *e *is a negative edge, *w*(*e*) = -1.

**Observation 1**. *There are at most O(n *· *m*^2^*) vertices and O(n *· *m*^2^*) edges in the pedigree graph*. Each member has *m *sites. The total number of vertices created from pairs of sites for each member is *O*(*m*^2^). The whole pedigree graph with *n *members has *O*(*n *· *m*^2^) vertices. A vertex has at most two positive edges linking it to two vertices in its parents. Therefore, the number of positive edges is linear in the number of vertices. The number of negative edges is also linear to the number of vertices. Thus the number of edges in the pedigree graph is *O*(*n *· *m*^2^).

### Parity-Constraint Sets

When a supplementary grey vertex *u_ij _*is created in *u *by the need of an adjacent member, there must be more than one grey vertex already created from site *i *to site *j *in *u*. It is important to ensure that these grey vertices and *u_ij _*when resolved will not result in an odd number of red vertices. Recall that a grey vertex is resolved red if *h*1*_u_*[*i*] ≠ *h*1*_u_*[*j*]. In other words, the value of *h*1*_u _*flips from 0 to 1 and vice versa for a red vertex *u_ij_*. Therefore there is a parity conflict if the number of red vertices from site *i *to site *j *including *u_ij _*is odd.

**Figure 3:**
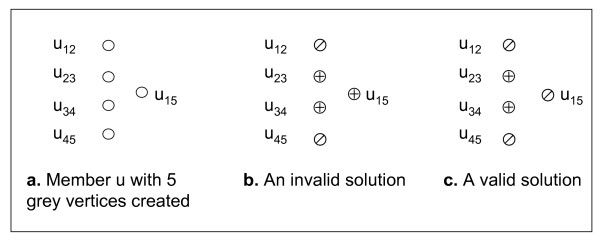
**Parity conflict between vertices within each member**.

In Figure 3a, there are five grey vertices created for member *u *where vertices *u*_12_, *u*_23_, *u*_34 _and *u*_45 _are created from closest heterozygous sites, and a supplementary vertex *u*_15 _is created for a member adjacent to *u*. Figure 3b shows an invalid solution with three resolved red vertices *u*_23_, *u*_34 _and *u*_15 _in member *u*. A valid solution with an even number of red vertices is shown in Figure 3c.

We create parity-constraint sets *S_pc _*to capture parity constraints between each supplementary vertex and other vertices within each member. Let *u_ij _*be a supplementary vertex and *u_ip_*, ..., *u_qj _*be grey vertices from site *i *to site *j*. These vertices form a parity-constraint set, and its total number of red vertices must be even. There are *O*(*m*^2^) parity-constraint sets in each member and *O*(*nm*^2^) parity-constraint sets for the whole pedigree graph. A valid solution for RHC*_k _*must ensure that the number of red vertices in each parity-constraint set is even.

## Signed Graph

A graph *G *= (*V*, *E*) is a *signed graph *if it has both positive and negative edges (*E *= *E_pos _*∪ *E_neg_*) [8], where *w*(*e_pos_*) = 1 and *w*(*e_neg_*) = - 1. Let (*V*_1_, *V*_2_) be a partition of *V *, and E* be the set of edges between *V*_1 _and *V*_2_. The *line index *of the cut (*V*_1_, *V*_2_) is defined as:(1)

The line index of graph G is defined as:(2)

The decision version of the line index of graph *G *is defined as follows.

**LineIndex***_k_*: *Given a signed graph G and a positive integer k, is there a line index of G at most k? *Given a pedigree graph *G *= (*V*, *E*), the RHC*_k _*problem can be solved by determining if we can label every grey vertex in *G *either red or green such that if we partition the set of vertices *V *into (*V_red_*, *V_green_*) and let E* be the set of edges between *V_red _*and *V_green _*then(3)

and this partition (*V_red_*, *V_green_*) must satisfy parity-constraint sets *S_pc_*.

Given a pedigree graph, any two adjacent members linked by a positive edge should be in the same set of the partition, and any two adjacent members linked by a negative edge should be in different sets. Any edge whose constraint is not satisfied represents a recombination event between the two adjacent members, or, in the case of a negative edge having endpoints in the same partition, between one parent and the child. Equation 3 thus counts the number of recombination events in the whole pedigree and ensures that it is at most *k*.

Clearly, the RHC*_k _*problem can be reduced to the LineIndex*_k _*problem with additional parity-constraint sets *S_pc _*on its vertices. We will show that the LineIndex*_k _*problem can be reduced to the GBER problem, a classic NP-complete problem that is fixed-parameter tractable. The RHC*_k _*can therefore be solved through the GBER problem with additional parity-constraint sets *S_pc_*.

**Theorem 1 ***A pedigree has at most k recombination events if and only if its corresponding signed graph has the line index of size at most k*.

**Proof 1 ***We will show that one recombination event in the pedigree corresponds to exactly one negative edge within each set of the partition of vertices or one positive edge between the sets of the partition of vertices in the signed graph*.

⇒ *Consider a recombination event in member u. To detect this recombination event there must be at least one heterozygous site on each side of the recombination breakpoint. Let i and j be the two closest heterozygous sites on the two sides of the recombination breakpoint. There are three possible types of vertices associated with this recombination event: a grey vertex u_ij_, an additional vertex u_ij'_, and supplementary vertices u_pq _*(*p *≤ *i*, *j *≤ *q*).

*If vertex u_ij _has an incident positive edge to a vertex c_ij_, the color u_ij _should be different from the color of c_ij _because of the recombination event and the positive edge between them would cross between sets of the partition. On the other hand, if u_ij _has an incident negative edge to a vertex v_ij_, the color u_ij _and v_ij _should be the same because of the recombination event and the negative edge between them would be within the same set of vertices. In both cases the line index increases by one. An additional vertex u_ij' _replaces u_ij _when u_ij _has no incident edge. The resolution of an additional vertex u_ij' _is similar to that of u_ij_. Consider a supplementary vertex u_pq _constrained by a parity-constraint set S_pc _where u_pq _has an incident positive edge to a vertex c_pq_. The color u_pq _is determined by the swap of values in h*1*_u _by red vertices and recombination events from p to q, including the recombination from i to j. If no more recombinations happen, u_pq _and c_pq _must have the same color and the line index of the signed graph is the same. If u_pq _and c_pq _have different colors, there must be another recombination from sites p to q and the line index increases by one. A similar explanation follows for u_pq _with an incident negative edge*.

⇐ *A negative edge links two vertices of two parents in a trio, and the two vertices are supposed to have different colors based on the Mendelian law of inheritance. Similarly, a positive edge links two vertices of a parent and a child and the two vertices are supposed to have the same color. Therefore, if a negative edge linking two vertices with the same color or a positive edge linking two vertices with different colors, one recombination event must happen*.

## Fixed-Parameter Algorithm

A NP-hard problem cannot be solved by a polynomial time algorithm unless P = NP. However, if we can restrict some parameters of the problem to small values, the running time of an algorithm for the problem can potentially be greatly reduced [10]. In this case, the problem is a *parameterized problem *and an algorithm that can solve the parameterized problem efficiently is a *fixed-parameter algorithm*, defined as follows [10].

**Definition 1 ***A parameterized problem is a language L *⊆ Σ* × Σ*, *where *Σ *is a finite alphabet and *Σ* *is the set of all strings over that alphabet. The second component is called the parameter of the problem*.

Practically, the parameter is a nonnegative integer or a set of nonnegative integers and therefore *L *⊆ Σ* × ℕ. For (*x*, *k*) ∈ *L*, the size of the input is *n *= |(*x*, *k*)|, and the parameter is *k*.

**Definition 2 ***A parameterized problem L is fixed-parameter tractable (in class FPT) if it can be determined in f(k)· n*^*O*(1) ^*time whether or not *(*x, k*) ∈ *L, where n is the size of the input and f is a computable function only depending on k*.

### Transforming to Bipartization by Edge Removal Problem

We review an important property of a signed graph given by [8].

**Theorem 2 ***Let G be a signed graph. If we replace each edge with weight w*(*e*) *>*0 *by two consecutive edges with weight -w*(*e*) *to get a graph G' then l*(*G*) *= l*(*G'*).

**Proof 2 ***Suppose *(*V*_1_, *V*_2_) *is a cut of G such that l*(*V_1_*, *V*_*2*_) *= l*(*G*)*. We replace each positive edge e(u*, *v) by two consecutive negative edges e(u*, *y) and e*(*y*, *v*), *where w(e(u*, *y*)) *= w(e(y*, *v*)) *= *- *w(e(u*, *v*)) *and y is a new vertex adjacent only to u and v. If u and v belong to the same set of vertices in the partition we put y into the other set. If u and v belong to different sets, we can arbitrarily put y into the same set as either u or v. In all of the cases above we find the corresponding cut of G'*, *such that *. *Therefore l(G') *≥ *l(G)*.

*Conversely, if **and y is a new vertex, then at least one edge incident to y is in the cut. We can find a corresponding cut of G*, (*V*_1_, *V*_2_) such that . *Therefore l*(*G') *≥ *l(G). Taken together, we get l*(*G'*) *= l*(*G*).

The pedigree graph is transformed into a new graph by replacing every positive edge by two consecutive negative edges and adding new intermediate vertices (*dum *vertices). We obtain a new weighted graph *G' *with all negative edges. This transformation does not affect the parity-constraint sets *S_pc_*. The graph *G' *still has only *O*(*n *· *m*^2^) vertices and *O*(*n *· *m*^2^) edges. Equation 3 becomes(4)

This equation is to ensure that the total number of edges within *V*_1 _and edges within *V*_2 _is at most *k*. Removing these edges will make the graph bipartite.

To make the GBER algorithm [9] works on our partially colored graph, we merge all red vertices into one red vertex and all green vertices into one green vertex. We relabel the merged red vertex and the merged green vertex into two grey vertices, and insert *k *+ 1 negative edges between them. This transformation does not affect the parity-constraint set *S_pc_*. We further transform our negative graph into a new graph with all positive edges by multiplying the weight of every edge by -1. Our problem becomes the GBER problem [9] with additional parity-constraint set *S_pc_*. The *k*-Bipartization by Edge Removal problem is defined as follows.

**Definition 3 ***Given a graph G *= (*V*, *E*) *and a positive integer k, is there a set C *⊆ *E with *|*C*| *≤ k whose removal produces a bipartite graph?*

GBER is a classical NP-hard problem [11] and is in FPT [9].

### FPT Algorithm for Bipartization by Edge Removal

There are many techniques to solve an FPT problem such as kernelization, depth-bounded search trees, dynamic programming, crown reduction, greedy localization, and iterative compression. The iterative compression technique is used by Guo et al. [9] to solve the GBER problem with a running time of *O*(2*^k ^*· |*E*|^2^), where |*E*| is the number of edge in the graph and *k *is the number of edges to be deleted to make the graph bipartite. However, this algorithm does not enforce our parity constraints that require the number of red vertices in each set to be even. We thus need to modify this algorithm [9] to solve the RHC*_k _*problem while respecting the additional parity-constraint sets *S_pc_*.

Given a graph *G *= (*V*, *E*) where *E *= {*e*_1_, ..., *e_m_*}, let *G_i _*be a graph induced by edges {*e*_1_, ..., *e_i_*} of *G *(1 ≤ *i *≤ *m*). If *i *= 1, the optimal edge bipartization set of *G*_1 _is empty. If *i *> 1, let *X *be an optimal edge bipartization set of *G_i _*= *G*[*e*_1_, ..., *e_i_*] and |*X*| = *k'*. Consider graph *G*_*i*+1 _= *G*[*e*_1_, ..., *e*_*i*+1_]. If *X *is not an optimal edge bipartization set for *G*_*i*+1 _then *X' *= *X *∪ {*e*_*i*+1_} is clearly an edge bipartization set for *G*_*i*+1_. From the edge bipartization set *X' *of size *k' *+ 1, we find an edge bipartization set of size at most *k' *or show that no such edge bipartization set of size at most *k' *exists. The algorithm assumes that an edge bipartization *Y *which is smaller than *X' *must be disjoint from *X'*, *Y *∩ *X' *= ∅. This assumption can be made without loss of generality by a simple graph transformation, replacing each edge in *X' *by three consecutive edges and choosing the middle edge to be in the new *X'*. This graph transformation preserves the parities of lengths of all cycles and does not affect the parity constraint sets *S_pc_*. Therefore the transformed graph has an edge bipartization set of size *k' *if and only if the original graph has an edge bipartization set of size *k'*. Let mapping Φ: *V *(*X'*) → {*A*, *B*} be a valid partition of *V *(*X'*) if for each {*y*, *z*} ∈ *X*, we have Φ(*y*) ≠ Φ(*z*). Let *A*_Φ _be Φ^-1^(*A*) and *B*_Φ _be Φ^-1^(*B*). We enumerate all 2*^k' ^*valid partitions Φ of *V *(*X'*). For each valid partition Φ we find a minimum edge cut *Y *in *G*\*X' *between *A*_Φ _and *B*_Φ_. In other words, we use *X' *to partially color *G *and from the partially colored graph we compute a smaller bipartization set *Y*. This compression step is the core of the algorithm.

**Theorem 3 **[9]*Consider a graph G = (V*, *E) and a minimal edge bipartization set X' for G. For a set of edges Y *⊆ *E with X' *∩ *Y = *∅, *the following are equivalent:*

*(1) Y is an edge bipartization set for G*.

*(2) There is a valid partition *Φ *for V *(*X'*) *such that Y is an edge cut in Gn\X' between A*_Φ _*= *Φ^-1^*(A) and B*_Φ _*= *Φ^-1^*(B)*.

**Figure 4:**
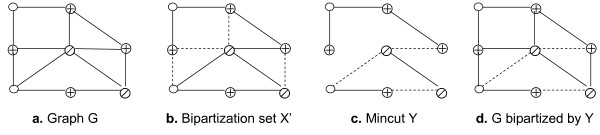
**Compression step**.

Consider a graph *G *in Figure 4a where ⊕ denotes a red vertex, ∅ a green vertex, and O a grey vertex. A minimal edge bipartization set *X' *of size 4 illustrated by dashed lines is given in Figure 4b. We compute a mincut *Y *for *G*\*X' *as in Figure 4c. Set *Y *is the edge bipartization set of size 3 for *G *in Figure 4d.

It remains to find a minimum edge cut *Y *between *A*_Φ _and *B*_Φ _that satisfies

(1) |*Y*| ≥ *k' *and

(2) graph *G_i _*with set *Y *satisfies parity-constraint sets *S_pc_*.

#### (s-t) Mincuts with parity constraints

A minimum edge cut *Y *between *A*_Φ _and *B*_Φ _can be computed in *O*(*k' *· |*E*|) time by the Edmonds-Karp algorithm [12] by finding at most *k' *augmenting paths; each path takes *O*(|*E*|) time to find. If no min edge cut *Y *of size *k' *is found, we skip the current partition Φ and check a new valid partition. If a min edge cut *Y *of size *k' *is found, we need to check if *G_i _*bipartized by *Y *satisfies the parity-constraint sets *S_pc_*. Note that there can be many mincuts *Y *of size *k' *between *A*_Φ _and *B*_Φ_, and it is possible that the current mincut *Y *found does not make *G_i _*satisfy *S_pc _*while another mincut *Y *of size *k' *makes *G_i _*satisfy *S_pc_*. However, enumerating all mincuts in a graph is expensive. Consider a simple directed graph with *n *disjoint paths of length 2 from a source *s *to a sink *t*, where the weight of each edge is 1. Each (s-t) mincut has weight *n *and we have up to 2*^n ^*(s-t) mincuts. If a graph is an undirected graph, we replace each undirected edge by two directed edges with opposite directions and the number of (s-t) mincuts is still 2*^n^*. Therefore enumerating all (s-t) mincuts in a graph in polynomial time, or in FPT, is impossible.

We do not enumerate all mincuts. Instead, we examine the structure of all mincuts in a graph by an algorithm in [13]. Given a graph *G *= (*V*, *E*) including a source *s *and a sink *t*, where each directed edge (*i*, *j*) ∈ *E *has a capacity *c_ij_*, an (s-t) cut (*S*, *S'*) is a cut where *S' *= *V *- *S*, *s *∈ *S *and *t *∈ *S'*. If a graph is not directed, we replace every undirected edge by two oppositely directed edges. If a graph has multiple sources and sinks, we can transform the graph into a new graph with only a single source and a single sink by inserting edges of ∞ weights from a *super source s *to all sources, and from all sinks into a *super sink t*. Flows and mincuts in the new and old graphs correspond [12].

An (s-t) mincut is an (s-t) cut where the total capacity of all the edges between *S *and *S' *is minimum. We will call an (s-t) mincut a mincut hereafter. Ford and Fulkerson [12] show that the value of a minimum cut between *s *and *t *is equal the value of the maximum flow from *s *to *t*. Consider a binary relation *R *on *V *, a subset of vertices *V' *⊆ *V *is a *closure *for *R *if and only if for any two vertices *i *and *j *in *V *with *iRj *and *i *∈ 2 *V' *we also have *j *∈ *V'*. Given a relation *iRj*, we say that *i *is the *predecessor *of *j *and *j *is a *successor *and *i*. Picard and Queyranne [13] present the relationship between mincuts and closures as follows.

**Theorem 4 **[13].

Let f be a maximum flow in G. Define a relation R on the set of vertices V as follows:

*iRj iff *(*i, j*) ∈ *E and f_ij _< c_ij_, or *(*j, i*) ∈ *E and f_ji _>*0.

*Then a cut (S, S') separating s from t is a minimum cut if an only if S is a closure for R containing s and not t*.

Suppose we find a maximum flow in a graph by the Edmonds-Karp algorithm [12]. Clearly, the residual graph *G_r _*= (*V*, *E_r_*) of *G *is defined by relation *R *where edge (*i*, *j*) ∈ *E_r _*iff *iRj*. We find strongly connected components in *G_r _*and shrink each of them into a single vertex. Finding strongly connected components of a directed graph *G_r _*can be done in *O*(*V *+ *E*) time using two depth first searches, one search on *G_r _*and the other search on the transpose graph  of *G_r _*[12].

Let *V' *be the reduced vertex set of *V *, we define a relation  on *V' *by  iff *iRj *for some , , and . We eliminate component *S *containing source *s *and its successor components, and eliminate component *T *containing sink *t *and its predecessor components. Combining *S *and all successor components with any closure induced from the remaining components will produce a mincut. When the number of sites *m *is small, we can check if a member can satisfy its parity-constraint sets by a backtracking search on at most *O*(*m*^2^) components. Since the parity constraints involve vertices for an individual member, these searches can be done independently. Therefore we need to examine if a valid partition Φ satisfies *S_pc _*on at most  cuts for the whole pedigree.

**Theorem 5 ***The RHC_k _problem is solvable in **time*.

**Proof 3 ***Setting up the pedigree graph G = (V, E) takes O*(|*V*|*) time, where *|*V*| *= *|*E| = O(nm^2^). Generating parity-constraint sets S_pc _takes O*(*nm^3^*)*. Transforming the pedigree graph into a graph with all negative edges takes O*(*|E|*) *time. The GBER problem can be solved by trying at most *2*^k ^valid partitions *Φ *. For each partition, we can find the first mincut in O*(*k ·|E|*) *time by finding at most k augmenting paths using Edmonds-Karp algorithm. We can find strongly connected components in O*(*|E|*) *time. We do backtracking in at most  cuts for each member to check if one can satisfy S_pc_; each check takes O*(*|E|*) *time. Therefore, checking each partition takes *. *The overall time complexity of the algorithm is *.

## Conclusion

We have shown that given a general pedigree with *n *members, *m *sites, and *k *recombination events, where *m *and *k *are small, the haplotype inference can be done in  time.

While not yet implemented, this algorithm should be implemented fairly easily. We only need to create a pedigree graph from input data according to the given construction and then transform the graph into the graph bipartization by edge removal with additional pedigree constraints, which can be tackled by making the appropriate modifications to an existing software package [14]. Future work will investigate the performance of the algorithm with simulated and real data.

## Competing interests

The authors declare that they have no competing interests.

## Authors' contributions

DDD designed the algorithm and drafted the manuscript. PAE supervised the research, assisted in crafting the algorithm and polished the manuscript. Both authors read and approved the final manuscript.
